# Tetra­aqua­(pyrimidine-4,6-dicarboxyl­ato-κ^2^ *N* ^1^,*O* ^6^)magnesium monohydrate

**DOI:** 10.1107/S1600536813005850

**Published:** 2013-03-06

**Authors:** Wojciech Starosta, Janusz Leciejewicz, Katarzyna Kiegiel

**Affiliations:** a Institute of Nuclear Chemistry and Technology, ul.Dorodna 16, 03-195 Warszawa, Poland

## Abstract

In the title compound, [Mg(C_6_H_2_N_2_O_4_)(H_2_O)_4_]·H_2_O, the Mg^II^ ion is coordinated by a fully deprotonated pyrimidine-4,6-dicarboxyl­ate mol­ecule, *via* a ring N and a carboxyl­ate O atom, and by four water O atoms at the apices of a slightly distorted octa­hedron. In the crystal, mol­ecules are linked by O—H⋯O and O—H⋯N hydrogen bonds, forming a three-dimensional network.

## Related literature

For the crystal structures of Mg complexes with pyrazine-2,3-dicarb­oxy­lic acid, see: Ptasiewicz-Bąk & Leciejewicz (1997 ▶), with pyrazine-2,5-dicarb­oxy­lic acid, see: Ptasiewicz-Bąk & Leciejewicz (1998 ▶), with pyrazine-2,6-dicarb­oxy­lic acid, see: Ptasiewicz-Bąk & Leciejewicz (2003 ▶) and with pyridazine-3,6-dicarb­oxy­lic acid, see: Gryz *et al.* (2004 ▶).
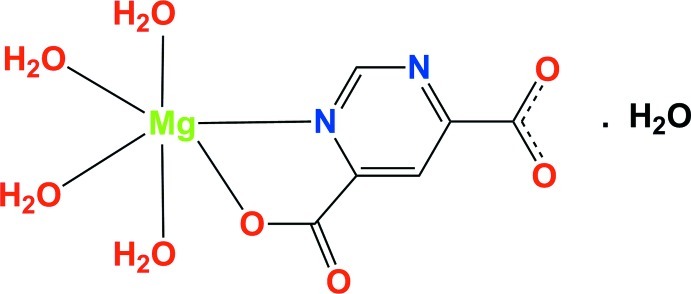


## Experimental

### 

#### Crystal data

[Mg(C_6_H_2_N_2_O_4_)(H_2_O)_4_]·H_2_O
*M* *_r_* = 280.49
Monoclinic, 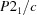

*a* = 7.5633 (15) Å
*b* = 6.7977 (14) Å
*c* = 21.605 (4) Å
β = 90.97 (3)°
*V* = 1110.6 (4) Å^3^
*Z* = 4
Mo *K*α radiation
μ = 0.21 mm^−1^
*T* = 293 K
0.25 × 0.23 × 0.09 mm

#### Data collection

Kuma KM-4 four-circle diffractometer
Absorption correction: analytical (*CrysAlis RED*; Oxford Diffraction, 2008 ▶) *T*_min_ = 0.947, *T*_max_ = 0.975
3485 measured reflections
3246 independent reflections
2187 reflections with *I* > 2σ(*I*)
*R*_int_ = 0.023
3 standard reflections every 200 reflections intensity decay: 5.10%

#### Refinement

*R*[*F* ^2^ > 2σ(*F* ^2^)] = 0.036
*wR*(*F* ^2^) = 0.125
*S* = 1.01
3246 reflections
203 parameters
H atoms treated by a mixture of independent and constrained refinement
Δρ_max_ = 0.47 e Å^−3^
Δρ_min_ = −0.26 e Å^−3^

### 

Data collection: *KM-4 Software* (Kuma, 1996 ▶); cell refinement: *KM-4 Software*; data reduction: *DATAPROC* (Kuma, 2001 ▶); program(s) used to solve structure: *SHELXS97* (Sheldrick, 2008 ▶); program(s) used to refine structure: *SHELXL97* (Sheldrick, 2008 ▶); molecular graphics: *SHELXTL* (Sheldrick, 2008 ▶); software used to prepare material for publication: *SHELXTL*.

## Supplementary Material

Crystal structure: contains datablock(s) I, global. DOI: 10.1107/S1600536813005850/su2567sup1.cif

Structure factors: contains datablock(s) I. DOI: 10.1107/S1600536813005850/su2567Isup2.hkl

Additional supplementary materials:  crystallographic information; 3D view; checkCIF report

## Figures and Tables

**Table 1:** Hydrogen-bond geometry (Å, °)

*D*—H⋯*A*	*D*—H	H⋯*A*	*D*⋯*A*	*D*—H⋯*A*
O8—H82⋯O2^i^	0.87 (3)	1.80 (3)	2.6640 (18)	171 (3)
O5—H51⋯O9^ii^	0.79 (3)	1.93 (3)	2.7102 (19)	170 (3)
O6—H61⋯N5^iii^	0.78 (4)	2.29 (4)	2.979 (2)	147 (3)
O6—H62⋯O3^iv^	0.90 (3)	1.88 (3)	2.7603 (19)	166 (3)
O8—H81⋯O4^iii^	0.86 (3)	1.93 (3)	2.779 (2)	174 (2)
O7—H71⋯O4^v^	0.90 (3)	1.78 (3)	2.6690 (18)	169 (3)
O5—H52⋯O7^vi^	0.77 (3)	2.09 (3)	2.8577 (19)	176 (3)
O9—H91⋯O1^vii^	0.77 (3)	2.02 (3)	2.7635 (18)	162 (4)
O9—H92⋯O3^iv^	0.81 (3)	1.91 (3)	2.6852 (18)	161 (3)
O7—H72⋯O9	0.85 (3)	1.85 (3)	2.6971 (19)	174 (3)

Symmetry codes: (i) 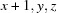; (ii) 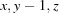; (iii) 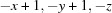; (iv) 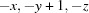; (v) 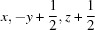; (vi) 
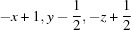
; (vii) 
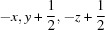
.

**Fig. 1.**
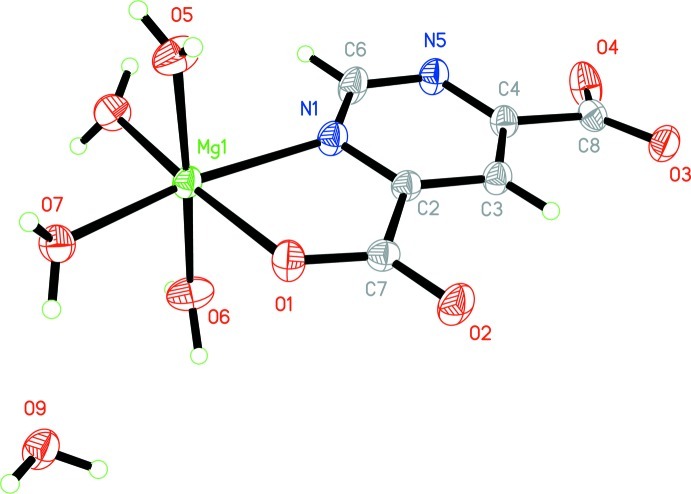
A view of the molecular structure of the title compound, with atom labelling. Displacement ellipsoids are drawn at the 50% probability level.

**Fig. 2.**
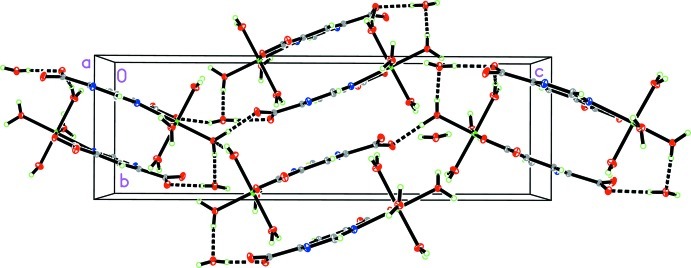
A view along the a axis of the crystal packing of the title compound. The hydrogen bonds are shown as dashed lines (see Table 1 for details).

## supplementary crystallographic information

### Comment

Crystal structures of Mg^II^ complexes with diazine dicarboxylate molecules belong to three types. For example, the structure of a Mg^II^ complex with pyrazine-2,3-dicarboxylate and water ligands is a catenated polymer (Ptasiewicz-Bąk & Leciejewicz, 1997), while those with pyrazine-2,5-dicarboxylate (Ptasiewicz-Bąk & Leciejewicz, 1998) and pyrazine-2,6-dicarboxylate (Ptasiewicz-Bąk & Leciejewicz, 2003) are composed of hexaquamagnesium(II) cations and fully deprotonated organic molecules.

On the other hand, the Mg^II^ complex with a pyridazine-3,6-dicarboxylate molecule is built of anions in which the Mg^II^ ion is coordinated by two water O atoms and two fully deprotonated organic molecules with singly protonated hydrazine molecules as cations (Gryz *et al.*, 2004).

The structure of the title compound, Fig. 1, is built of monomeric molecules in which a Mg^II^ ion is coordinated by one of the N,*O* bonding groups of a fully deprotonated pyrimidine-4,6-dicarboxylate molecule and four water O atoms. The coordination geometry of atom Mg1 is a slightly distorted octahedron with typical Mg-N and Mg—O distances [Mg1-N1 = 2.2472 (15) Å; the Mg1-O distances vary from 2.0120 (13) to 2.0896 (15) Å]. The carboxylic groups C7/O1/O2 and C8/O3/O4 are inclined to the pyrimidine ring by 3.5 (1)° and 14.9 (2)°, respectively.

In the crystal, the complexes interact *via* an extended network of O-H···O and O-H···N hydrogen bonds, in which coordinated and solvate water molecules are donors and the carboxylato O and hetero-ring N atoms act as acceptors, forming a three-dimensional network (Fig. 2 and Table 1).

### Experimental

An aqueous solution containing 1 mmol of magnesium acetate tetrahydrate and 1 mmol of pyrimidine-4,6-dicarboxylic acid dihydrate were refluxed with constant stirring for 2 h yielding a white precipitate which subsequently was filtered and redissolved in an excess of boiling water. Cooled to room temperature, the solution was left to evaporate. Colourless plate-like crystals deposited after a few days. They were washed with cold ethanol and dried in the air.

### Refinement

Water H atoms were located in a difference Fourier map and freely refined. The C-bound H atoms were positioned at calculated positions and treated as riding on the parent atoms: C—H = 0.93 Å with *U*_iso_(H )= 1.2*U*_eq_(C).

### Crystal data

**Table:**

[Mg(C_6_H_2_N_2_O_4_)(H_2_O)_4_]·H_2_O	*F*(000) = 584
*M**_r_* = 280.49	*D*_x_ = 1.677 Mg m^−^^3^
Monoclinic, *P*2_1_/*c*	Mo *K*α radiation, λ = 0.71073 Å
Hall symbol: -P 2ybc	Cell parameters from 25 reflections
*a* = 7.5633 (15) Å	θ = 6–15°
*b* = 6.7977 (14) Å	µ = 0.21 mm^−^^1^
*c* = 21.605 (4) Å	*T* = 293 K
β = 90.97 (3)°	Plates, colourless
*V* = 1110.6 (4) Å^3^	0.25 × 0.23 × 0.09 mm
*Z* = 4	

### Data collection

**Table:**

Kuma KM-4 four-circle diffractometer	2187 reflections with *I* > 2σ(*I*)
Radiation source: fine-focus sealed tube	*R*_int_ = 0.023
Graphite monochromator	θ_max_ = 30.1°, θ_min_ = 1.9°
profile data from ω/2θ scans	*h* = −10→0
Absorption correction: analytical (*CrysAlis RED*; Oxford Diffraction, 2008)	*k* = 0→9
*T*_min_ = 0.947, *T*_max_ = 0.975	*l* = −30→30
3485 measured reflections	3 standard reflections every 200 reflections
3246 independent reflections	intensity decay: 5.1%

### Refinement

**Table:**

Refinement on *F*^2^	Primary atom site location: structure-invariant direct methods
Least-squares matrix: full	Secondary atom site location: difference Fourier map
*R*[*F*^2^ > 2σ(*F*^2^)] = 0.036	Hydrogen site location: inferred from neighbouring sites
*wR*(*F*^2^) = 0.125	H atoms treated by a mixture of independent and constrained refinement
*S* = 1.01	*w* = 1/[σ^2^(*F*_o_^2^) + (0.0833*P*)^2^ + 0.2045*P*] where *P* = (*F*_o_^2^ + 2*F*_c_^2^)/3
3246 reflections	(Δ/σ)_max_ < 0.001
203 parameters	Δρ_max_ = 0.47 e Å^−^^3^
0 restraints	Δρ_min_ = −0.26 e Å^−^^3^

### Special details

**Table:**

Geometry. All e.s.d.'s (except the e.s.d. in the dihedral angle between two l.s. planes) are estimated using the full covariance matrix. The cell e.s.d.'s are taken into account individually in the estimation of e.s.d.'s in distances, angles and torsion angles; correlations between e.s.d.'s in cell parameters are only used when they are defined by crystal symmetry. An approximate (isotropic) treatment of cell e.s.d.'s is used for estimating e.s.d.'s involving l.s. planes.
Refinement. Refinement of *F*^2^ against ALL reflections. The weighted *R*-factor *wR* and goodness of fit *S* are based on *F*^2^, conventional *R*-factors *R* are based on *F*, with *F* set to zero for negative *F*^2^. The threshold expression of *F*^2^ > σ(*F*^2^) is used only for calculating *R*-factors(gt) *etc*. and is not relevant to the choice of reflections for refinement. *R*-factors based on *F*^2^ are statistically about twice as large as those based on *F*, and *R*- factors based on ALL data will be even larger.

### Fractional atomic coordinates and isotropic or equivalent isotropic displacement parameters (Å^2^)

**Table:**

	*x*	*y*	*z*	*U*_iso_*/*U*_eq_	
Mg1	0.32572 (6)	0.46078 (8)	0.15804 (2)	0.01813 (14)	
O1	0.05876 (14)	0.42580 (19)	0.14674 (5)	0.0237 (2)	
O3	0.00597 (16)	0.06801 (19)	−0.12636 (5)	0.0273 (3)	
H82	0.667 (4)	0.431 (4)	0.1337 (12)	0.043 (7)*	
O2	−0.16427 (15)	0.3374 (2)	0.08385 (6)	0.0284 (3)	
O5	0.36240 (18)	0.1989 (2)	0.20187 (7)	0.0304 (3)	
O7	0.30383 (16)	0.59579 (19)	0.24329 (5)	0.0236 (2)	
N1	0.29679 (17)	0.3154 (2)	0.06501 (6)	0.0211 (3)	
C7	−0.00682 (19)	0.3566 (2)	0.09709 (7)	0.0188 (3)	
C2	0.12644 (18)	0.2956 (2)	0.04897 (7)	0.0179 (3)	
C3	0.07484 (19)	0.2275 (2)	−0.00879 (7)	0.0200 (3)	
H3	−0.0439	0.2128	−0.0196	0.024*	
C4	0.2075 (2)	0.1819 (2)	−0.04993 (7)	0.0195 (3)	
O6	0.30977 (19)	0.7335 (2)	0.11370 (7)	0.0312 (3)	
O4	0.28320 (19)	0.1211 (2)	−0.15411 (6)	0.0355 (3)	
C8	0.1616 (2)	0.1171 (2)	−0.11589 (7)	0.0217 (3)	
N5	0.37836 (17)	0.1979 (2)	−0.03413 (6)	0.0242 (3)	
C6	0.41441 (19)	0.2628 (3)	0.02291 (8)	0.0247 (3)	
H6	0.5331	0.2722	0.0344	0.030*	
O9	0.12147 (16)	0.9368 (2)	0.24311 (6)	0.0267 (3)	
H51	0.290 (4)	0.132 (5)	0.2170 (13)	0.052 (8)*	
H61	0.389 (5)	0.794 (5)	0.1006 (15)	0.067 (10)*	
H62	0.216 (4)	0.815 (4)	0.1146 (12)	0.046 (7)*	
H81	0.629 (3)	0.613 (4)	0.1576 (11)	0.040 (7)*	
O8	0.58966 (15)	0.4954 (2)	0.15529 (6)	0.0264 (3)	
H71	0.282 (4)	0.526 (4)	0.2778 (13)	0.043 (7)*	
H52	0.452 (4)	0.166 (5)	0.2159 (13)	0.050 (8)*	
H91	0.069 (4)	0.959 (5)	0.2725 (15)	0.063 (9)*	
H92	0.061 (4)	0.939 (5)	0.2119 (14)	0.052 (8)*	
H72	0.241 (4)	0.699 (4)	0.2451 (11)	0.038 (7)*	

### Atomic displacement parameters (Å^2^)

**Table:**

	*U*^11^	*U*^22^	*U*^33^	*U*^12^	*U*^13^	*U*^23^
Mg1	0.0142 (2)	0.0234 (3)	0.0168 (2)	−0.00041 (18)	0.00078 (17)	−0.00113 (18)
O1	0.0172 (5)	0.0342 (6)	0.0199 (5)	−0.0025 (4)	0.0039 (4)	−0.0066 (4)
O3	0.0225 (5)	0.0341 (6)	0.0251 (6)	0.0008 (5)	−0.0041 (4)	−0.0043 (5)
O2	0.0139 (5)	0.0429 (7)	0.0284 (6)	0.0009 (5)	0.0011 (4)	−0.0072 (5)
O5	0.0223 (6)	0.0293 (7)	0.0396 (7)	−0.0006 (5)	−0.0016 (5)	0.0119 (5)
O7	0.0266 (5)	0.0260 (6)	0.0183 (5)	0.0014 (5)	0.0024 (4)	0.0000 (5)
N1	0.0157 (5)	0.0284 (7)	0.0192 (6)	−0.0001 (5)	0.0009 (4)	−0.0028 (5)
C7	0.0147 (6)	0.0219 (7)	0.0198 (6)	0.0009 (5)	0.0025 (5)	−0.0009 (5)
C2	0.0148 (6)	0.0204 (7)	0.0185 (6)	0.0000 (5)	0.0014 (5)	−0.0007 (5)
C3	0.0158 (6)	0.0248 (7)	0.0193 (7)	−0.0018 (5)	0.0010 (5)	−0.0024 (5)
C4	0.0199 (6)	0.0223 (7)	0.0165 (6)	−0.0017 (5)	0.0015 (5)	−0.0018 (5)
O6	0.0238 (6)	0.0307 (6)	0.0394 (7)	0.0042 (5)	0.0085 (5)	0.0103 (5)
O4	0.0368 (7)	0.0463 (8)	0.0238 (6)	−0.0141 (6)	0.0110 (5)	−0.0125 (6)
C8	0.0257 (7)	0.0215 (7)	0.0178 (6)	−0.0013 (6)	0.0006 (5)	−0.0028 (5)
N5	0.0170 (6)	0.0340 (8)	0.0217 (6)	−0.0011 (5)	0.0021 (5)	−0.0051 (5)
C6	0.0134 (6)	0.0383 (9)	0.0225 (7)	0.0000 (6)	0.0009 (5)	−0.0052 (6)
O9	0.0203 (5)	0.0387 (7)	0.0210 (6)	0.0009 (5)	0.0008 (4)	0.0008 (5)
O8	0.0154 (5)	0.0310 (6)	0.0329 (6)	−0.0015 (4)	0.0042 (4)	−0.0036 (5)

### Geometric parameters (Å, º)

**Table:**

Mg1—O8	2.0120 (13)	C7—C2	1.518 (2)
Mg1—O5	2.0331 (15)	C2—C3	1.381 (2)
Mg1—O1	2.0436 (13)	C3—C4	1.387 (2)
Mg1—O7	2.0671 (13)	C3—H3	0.9300
Mg1—O6	2.0896 (15)	C4—N5	1.335 (2)
Mg1—N1	2.2472 (15)	C4—C8	1.526 (2)
O1—C7	1.2650 (19)	O6—H61	0.78 (4)
O3—C8	1.241 (2)	O6—H62	0.90 (3)
O2—C7	1.2270 (19)	O4—C8	1.247 (2)
O5—H51	0.79 (3)	N5—C6	1.333 (2)
O5—H52	0.77 (3)	C6—H6	0.9300
O7—H71	0.90 (3)	O9—H91	0.77 (3)
O7—H72	0.85 (3)	O9—H92	0.81 (3)
N1—C6	1.332 (2)	O8—H82	0.87 (3)
N1—C2	1.3353 (19)	O8—H81	0.86 (3)
			
O8—Mg1—O5	89.34 (6)	O2—C7—C2	117.67 (13)
O8—Mg1—O1	171.45 (6)	O1—C7—C2	115.28 (13)
O5—Mg1—O1	94.62 (6)	N1—C2—C3	121.66 (13)
O8—Mg1—O7	93.95 (6)	N1—C2—C7	116.35 (13)
O5—Mg1—O7	89.20 (6)	C3—C2—C7	121.98 (13)
O1—Mg1—O7	93.69 (6)	C2—C3—C4	117.23 (13)
O8—Mg1—O6	86.12 (6)	C2—C3—H3	121.4
O5—Mg1—O6	175.43 (6)	C4—C3—H3	121.4
O1—Mg1—O6	89.95 (6)	N5—C4—C3	121.71 (14)
O7—Mg1—O6	90.56 (6)	N5—C4—C8	117.78 (13)
O8—Mg1—N1	96.12 (6)	C3—C4—C8	120.49 (13)
O5—Mg1—N1	92.41 (6)	Mg1—O6—H61	126 (3)
O1—Mg1—N1	76.17 (5)	Mg1—O6—H62	125.1 (17)
O7—Mg1—N1	169.82 (5)	H61—O6—H62	107 (3)
O6—Mg1—N1	88.64 (6)	O3—C8—O4	126.41 (15)
C7—O1—Mg1	121.14 (10)	O3—C8—C4	116.62 (14)
Mg1—O5—H51	128 (2)	O4—C8—C4	116.96 (14)
Mg1—O5—H52	123 (2)	C6—N5—C4	116.43 (13)
H51—O5—H52	106 (3)	N1—C6—N5	126.29 (14)
Mg1—O7—H71	121.5 (17)	N1—C6—H6	116.9
Mg1—O7—H72	117.5 (17)	N5—C6—H6	116.9
H71—O7—H72	107 (2)	H91—O9—H92	113 (3)
C6—N1—C2	116.64 (13)	Mg1—O8—H82	129.2 (18)
C6—N1—Mg1	132.29 (11)	Mg1—O8—H81	117.0 (18)
C2—N1—Mg1	110.81 (10)	H82—O8—H81	105 (3)
O2—C7—O1	127.04 (14)		

### Hydrogen-bond geometry (Å, º)

**Table:**

*D*—H···*A*	*D*—H	H···*A*	*D*···*A*	*D*—H···*A*
O8—H82···O2^i^	0.87 (3)	1.80 (3)	2.6640 (18)	171 (3)
O5—H51···O9^ii^	0.79 (3)	1.93 (3)	2.7102 (19)	170 (3)
O6—H61···N5^iii^	0.78 (4)	2.29 (4)	2.979 (2)	147 (3)
O6—H62···O3^iv^	0.90 (3)	1.88 (3)	2.7603 (19)	166 (3)
O8—H81···O4^iii^	0.86 (3)	1.93 (3)	2.779 (2)	174 (2)
O7—H71···O4^v^	0.90 (3)	1.78 (3)	2.6690 (18)	169 (3)
O5—H52···O7^vi^	0.77 (3)	2.09 (3)	2.8577 (19)	176 (3)
O9—H91···O1^vii^	0.77 (3)	2.02 (3)	2.7635 (18)	162 (4)
O9—H92···O3^iv^	0.81 (3)	1.91 (3)	2.6852 (18)	161 (3)
O7—H72···O9	0.85 (3)	1.85 (3)	2.6971 (19)	174 (3)

Symmetry codes: (i) *x*+1, *y*, *z*; (ii) *x*, *y*−1, *z*; (iii) −*x*+1, −*y*+1, −*z*; (iv) −*x*, −*y*+1, −*z*; (v) *x*, −*y*+1/2, *z*+1/2; (vi) −*x*+1, *y*−1/2, −*z*+1/2; (vii) −*x*, *y*+1/2, −*z*+1/2.

## References

[bb1] Gryz, M., Starosta, W. & Leciejewicz, J. (2004). *J. Coord. Chem.* **57**, 917–922.

[bb2] Kuma (1996). *KM-4 Software* Kuma Diffraction Ltd. Wrocław, Poland.

[bb3] Kuma (2001). *DATAPROC* Kuma Diffraction Ltd. Wrocław, Poland.

[bb4] Oxford Diffraction (2008). *CrysAlis RED* Oxford Diffraction Ltd., Yarnton, England.

[bb5] Ptasiewicz-Bąk, H. & Leciejewicz, J. (1997). *Pol. J. Chem.* **71**, 493–500.

[bb6] Ptasiewicz-Bąk, H. & Leciejewicz, J. (1998). *J. Coord. Chem.* **44**, 299–309.

[bb7] Ptasiewicz-Bąk, H. & Leciejewicz, J. (2003). *J. Coord. Chem.* **56**, 173–180.

[bb8] Sheldrick, G. M. (2008). *Acta Cryst.* A**64**, 112–122. 10.1107/S0108767307043930 18156677

